# Comparative Transcriptome Analysis Provides Insight into Spatio-Temporal Expression Characteristics and Genetic Regulatory Network in Postnatal Developing Subcutaneous and Visceral Fat of Bama Pig

**DOI:** 10.3389/fgene.2022.844833

**Published:** 2022-03-31

**Authors:** Yingying Zhang, Hongyang Wang, Weilong Tu, Sayed Haidar Abbas Raza, Jianguo Cao, Ji Huang, Huali Wu, Chun Fan, Shengchang Wang, Ying Zhao, Yongsong Tan

**Affiliations:** ^1^ Institute of Animal Husbandry and Veterinary Science, Shanghai Academy of Agricultural Sciences, Shanghai, China; ^2^ Shanghai Engineering Research Center of Breeding Pig, Shanghai, China; ^3^ College of Animal Science and Technology, Northwest A&F University, Yangling, China; ^4^ Shanghai Laboratory Animal Research Center, Shanghai, China

**Keywords:** Bama pig, subcutaneous fat, visceral fat, transcriptome analysis, insulin response, organ size regulation

## Abstract

The depot differences between Subcutaneous Fat (SAF) and Visceral Fat (VAF) are critical for human well-being and disease processes in regard to energy metabolism and endocrine function. Miniature pigs (*Sus scrofa*) are ideal biomedical models for human energy metabolism and obesity due to the similarity of their lipid metabolism with that of humans. However, the regulation of differences in fat deposition and development remains unclear. In this study, the development of SAF and VAF was characterized and compared in Bama pig during postnatal development (infancy, puberty and adulthood), using RNA sequencing techniques (RNA-Seq). The transcriptome of SAF and VAF was profiled and isolated from 1-, 3- and 6 months-old pigs and identified 23,636 expressed genes, of which 1,165 genes were differentially expressed between the depots and/or developmental stages. Upregulated genes in SAF showed significant function and pathway enrichment in the central nervous system development, lipid metabolism, oxidation-reduction process and cell adhesion, whereas genes involved in the immune system, actin cytoskeleton organization, male gonad development and the hippo signaling pathway were preferentially expressed in VAF. Miner analysis of short time-series expression demonstrated that differentiation in gene expression patterns between the two depots corresponded to their distinct responses in sexual development, hormone signaling pathways, lipid metabolism and the hippo signaling pathway. Transcriptome analysis of SAF and VAF suggested that the depot differences in adipose tissue are not only related to lipid metabolism and endocrine function, but are closely associated with sexual development and organ size regulation.

## Introduction

There is an increasing understanding that adipose tissue contributes to both human well-being and disease processes (Pradat, 2021). Moderate amounts of adipose tissue are essential for health. In addition to its main function in thermogenesis, in energy storage, release and regulation, and in homeostasis, research has shown the potential beneficial effects of adipose tissue in the evolution of the human brain (Aiello and Wheeler, 1995). On the other hand, excess fat mass in body can be harmful. Adipose tissue development results in obesity and related comorbidities; it is an important endocrine organ that secretes a number of pro- and anti-inflammatory adipokines, which can increase the risk for multiple metabolic diseases, such as type 2 diabetes, cardiovascular disease and cancer (Lee et al., 2013; Pradat, 2021).

According to adipose tissue anatomy and distribution, these can be divided into two main categories, subcutaneous fat (SAF) and visceral fat (VAF), which are intrinsically different as a result of genetic or developmental events. Heterogeneity among SAF and VAF include depot differences in morphology and cellular composition (Van Harmelen et al., 2004; Tchkonia et al., 2005), metabolism (Wang et al., 2003; Bartness and Song, 2007; Fried et al., 2010; Koutsari et al., 2011) and production (Varma et al., 2007; Harvey et al., 2020), which collectively form the “microenvironment” within each depot contributing to differences in metabolism and endocrine function (Pradat, 2021). For instance, the number of stromal cells per gram of tissue is fewer in abdominal SAF than in omental VAF (Van Harmelen et al., 2004), but contains greater numbers of preadipocytes in SAF (Tchkonia et al., 2005; Karpe and Pinnick, 2015). Expression of hormone-sensitive lipase (HSL) and perilipinn, two lipid droplet-associated proteins, are differentially expressed between SAF and VAF (Wang et al., 2003), glucose uptake in VAF being greater than in SAF (Virtanen et al., 2002). Expression of proinflammatory cytokines (interleukin-6, IL-6, interleukin-8, IL-8, and macrophage chemoattractant protein-1, MCP-1) were generally higher in VAF, whereas leptin and IP-10 expression was higher in SAT (Harvey et al., 2020). Moreover, molecules that are involved in innate immunity, acute phase response and complement factors are overexpressed in VAT (Madani et al., 2009). However, the molecular mechanism of heterogeneity between SAF and VAF is not completely clear so that further research is needed. The factors affecting adipose tissue distribution include race, sex, age and other factors (Pradat, 2021). Age has been considered one of the complex factors determining fatness and fat distribution. Fat accumulates in the central area of both SAF and VAF during aging (Kuk et al., 2009), and there is evidence showing that obesity increases risk for morbidity and mortality in young people, whereas its effect in the elderly is much more complex (Bosello and Vanzo, 2021). Nevertheless, the mechanisms involved are poorly understood.

The domestic pig (*Sus scrofa*), particularly the miniature breeds, has served widely as an important model organism for studies of human disease and comparative genome studies, not only because swine are similar in organ size, anatomical structure, physiological indicators, organ development and disease progression with those of humans, but because of their high genomic sequence and chromosome structure homology with humans (Rothschild, 2004; Schook et al., 2005; Groenen et al., 2012; Alföldi and Lindblad-Toh, 2013). Adipocyte biology is pivotal in the regulation of fat metabolism, glucose absorption, energy metabolism and innate immune responses in pigs (Lunney, 2007; Gabler et al., 2009). Differential deposition and its regulatory mechanisms between SAF and intramuscular fat (IMF) have been proved (Tous et al., 2013; Wu et al., 2020). Bama pig, a Chinese indigenous miniature pig breed inhabiting the Guangxi province, is being increasingly developed as an animal model for medical research, for example, in human drug evaluation, xenotransplantation and a range of diseases, and the development of induced pluripotent stem (Ips) cell lines (Li et al., 2006; Liu et al., 2008; Chen et al., 2009; Esteban et al., 2009; Liu et al., 2009; Liu et al., 2010; Zhang et al., 2011; Zhang et al., 2012). Although they have a crucial role, our knowledge of patterns of transcriptome divergence between different postnatal developmental stages within and between SAF and VAF are poorly studied.

To help improve this situation, we have examined gene expression profiles in SAF and VAF of pigs during postnatal development using RNA sequencing techniques (RNA-Seq). This allowed us to identify the differential dynamics of gene expression of SAF and VAF, and investigate the underlying functional differences in differentially expressed genes (DEGs) during postnatal development, and between the two depots, and should improve our understanding of SAF and VAF deposition.

## Material and Methods

### Animal and Ethics Statements

Animals used were conducted according to the Regulations for the Administration of Affairs Concerning Experimental Animals (Ministry of Science and Technology, China, June 2004) and approved by the Institutional Animal Care and Use Committee (IACUC) of Shanghai Academy of Agricultural Sciences (approved ID: SAASPZ0520012, Shanghai, China, 24 December 2020). Male Bama pigs with similar genetic backgrounds produced by one sire were born and raised under the same environmental and nutritional conditions, housed at Taizhou experimental miniature pig breeding case managed by Taihe Biotechnology Co., Ltd., in Taizhou, Jiangsu Province, China. Animals were fasted 24 h prior to slaughter. Fasting blood samples were collected. Animals were then euthanized with pentobarbital sodium (150 mg/kg). The adipose tissues were sampled immediately after death.

### Biological Material and RNA-Seq Experiments

Nine Bama male pigs were included in this study 3 groups, 3 of them were 1 months old (infancy, weight = 4.70 ± 0.17 kg, 20 October 2019), 3 being 3 months-old (puberty, weight = 7.57 ± 0.32 kg, 18 August 2019), and 3 were 6 months-old (adulthood, weight = 17.83 ± 3.01 kg, 12 May 2019). SAF on the back between the third and fourth last ribs and VAF adjacent to kidney deposition were isolated from pigs and frozen immediately in liquid nitrogen until lipid extraction. A portion of fresh SAF and VAF were cut into about a cubic centimeter of tissue and fixed in 4% paraformaldehyde for histological analysis.

Total RNA was extracted from adipose tissues using TRIzol reagent (Invitrogen, Carlsbad, CA, United States). The samples were purified using a Rneasy Mini Kit (Qiagen, Hilden, Germany). RNA integrity was assessed with a 2200 Bioanalyzer (Agilent Technologies, Inc., Santa Clara, CA, United States) and agarose gel electrophoresis.

RNA integrity number (RIN) of the samples range from 6.6 to 9.4. The RNA sequence libraries were prepared with a TruSeq RNA Sample Preparation Kit (Illumina, San Diego, CA, United States). The quality of Cdna libraries was checked using an Agilent 2200 TapeStation system (Agilent, Palo Alto, CA, United States). The cDNA libraries were sequenced with an Illumina HiSeq X ten Illumina, San Diego, CA, United States which generated paired-end raw reads of approximately 150 bp in size. The fastq-formatted RNA-seq datasets have been deposited in the National Center for Biotechnology Information (NCBI) Gene Expression Omnibus database (GEO: GSE184038).

The fastp were used for removing the adaptor sequence and discarding the low-quality reads to achieve the clean data (Chen et al., 2018). A read will be discarded when it does not meet requirements of the following parameters, reads length ≥50 bp, the Nbase number ≤5 and less than 40 percent of bases are unqualified (quality score ≥ 15). The clean reads were aligned to 9,823(NCBI Taxonomy ID)genomes (version: sscrofa11.1) using the Hisat2 (Kim et al., 2019). The RNA-seq count was calculated by Htseq-count and the expression data were normalized using fragments per kilobase of exon model per Million mapped fragments (FPKM). The DEGs were filtered by using DESeq2 algorithm under the following criteria: 1) log2FC >0.585 or <−0.585; ii), FDR <0.05 (Love et al., 2014)

### Functional Enrichment Analysis

ClusterProfiler was used for GO and Pathway enrichment (Yu et al., 2012). We downloaded the GO annotations from the Gene Ontology (http://www.geneontology.org/) and Pathway annotation from Kyoto Encyclopedia of Genes and Genomes (KEGG) (https://www.kegg.jp/). The *p*-value can be calculated by hypergeometric distribution, which corresponded to a one-sided version of Fisher’s exact test. FDR was used to correct *p*-values (*p*-values < 0.05) (Ashburner et al., 2000). GO and Pathway enrichment was also applied to the genes belonging to specific profiles. Significant profiles were identified using Fisher’s exact test and multiple comparisons.

### QRT-PCR

QRT-PCR were analyzed using an Applied Biosystems StepOnePlus Real-Time PCR System (Applied Biosystems, Foster City, CA, United States). Total RNA from SAF and VAF tissues were isolated using TRIzol^®^ Reagent (Invitrogen, Carlsbad, CA, United States); cDNAs were synthesized using a Thermo Scientific RevertAid First Strand Cdna Synthesis Kit; and QRT-PCR analyses were carried by using Roche FastStart Universal SYBR Green Master (Rox). Mrna primers were designed using Primer 5.0 software (Primer-E Ltd., Plymouth, United Kingdom) and their sequences are listed in Supplementary Tables S1–S2. Parallel reactions using *GAPDH* were performed to normalize the amount of templated cDNA. Each of the amplifications was triplicated and the mean value was calculated using the △△C*t* method. The results (FC) were expressed as 2^△△C*t*^: $△△Ct=\left(Ct_{ij}-Ct_{GAPDHj}\right)-\left(Ct_{i1}-Ct_{GAPDH1}\right)$ , where C*t*_ij_ and C*t*_GAPDHj_ are the C*t* values for gene I and for GAPDH in a sample (named j); Ct_i1_ and Ct_GAPDH1_ are the Ct values in sample 1, expressed as the standard. A Student’s *t*-test of independent data was used to assess the statistical significance of differential expression levels of each gene within the samples.

### Short Time-Series Expression Miner Analysis

STEM analysis is a commonly used bioinformatics method used to determine statistically significant time-dependent gene expression profiles (Ernst and Bar-Joseph, 2006). Expression profiles of DEGs were determined by cluster analysis based on the STEM method (http://www.cs.cmu.edu/∼jernst/st/) (Draghici et al., 2007; Ehrenkaufer et al., 2013). it assumes the values of gene expression represent log ratios relative to the expression at the first time-point. The approach selects a set of predetermined temporal model profiles and determines the statistical significance of the number of genes assigned to each profile compared to the number of genes expected based on chance. This method was used to explore mRNAs that might be correlated with fat development. Significant profiles were identified using Fisher’s exact test and multiple comparisons. GO and Pathway enrichment was also applied to the genes belonging to specific profiles.

### Construction of Molecular Interaction Network

One of the advanced bioinformatic tools, Ingenuity Pathway Analysis (IPA) software program was used to construct a molecular interaction network for better interpretation of the gene expression profiles as revealed by RNA-seq on the strength of a build-in scientific literature-based database (IPA Ingenuity Web Site, www.ingenuity.com), a very convenient software program to classify the pathways, molecular networks and functions most relevant to genes of interest or experimental datasets (Calvano et al., 2005; Mayburd et al., 2006).

## Results

### Genome-Wide Identification of the Expressed Genes in Subcutaneous Fat and Visceral Fat

**FIGURE 1:**
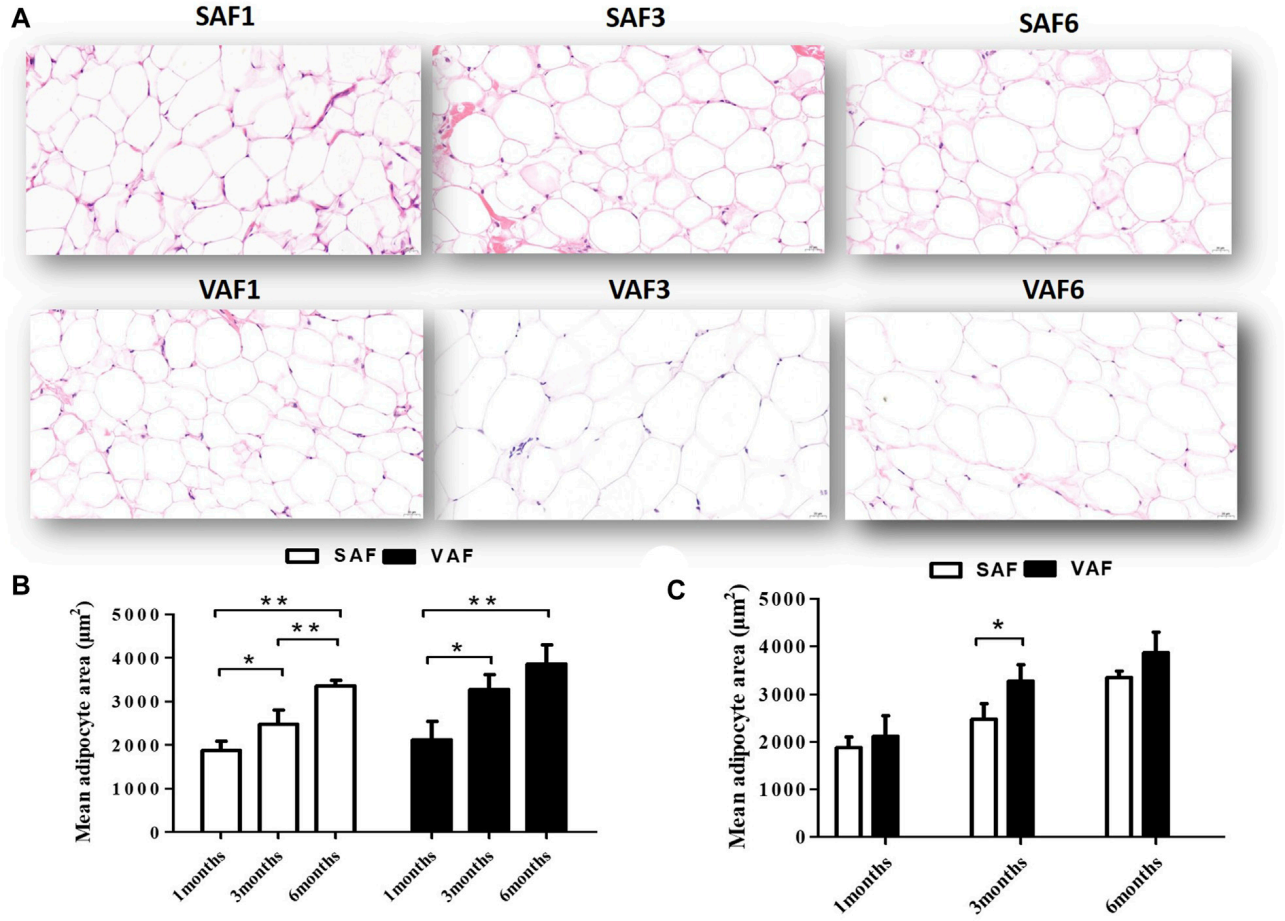
Histological sections of SAF tissue and VAF tissue of pigs during the postnatal stage. **(A)**. H&E staining of SAF tissue and VAF tissue at 1, 3 and 6 months (x400 magnification, scale bar: 20μm); **(B,C)**. Mean adipocyte size of SAF and VAF tissues at the postnatal stage; areas were measured by Images J software for 3 different animals per group (0.122 mm^2^ total area for each sample). ^*^ *p* < 0.05, ^**^ *p* < 0.01.

Nine male pigs were included in this study measuring the body fatness traits at 1, 3 and 6 months of age (Supplementary Table S3). At 6 months, all measured indicators were significantly higher than those at 1 and 3 months (*p* < 0.05). Body fat rate, backfat thickness, perirenal, mesenteric fat and greater omentum weights at 3 months were significantly higher than at 1 month (*p* < 0.05), but weights were similar between 1 and 3 months (*p* > 0.05). This corresponds to the larger adipocyte size in pigs at 1 month compared with 3 and 6 months (Figures 1A–C). Histological examination showed that size significantly increased during the postnatal stage for both SAF and VAF (*p* < 0.05, *p* < 0.01). In addition, sizes in VAF were significantly bigger than SAF at 3 months (*p* < 0.05), whereas differences were insignificant at 1 and 6 months (*p* > 0.05). We hypothesize that SAF and VAF in the pigs were systemically differentiating at 3 months and fat development was distinct between the depositions of SAF and VAF. On this basis, we investigated the SAF and VAF transcriptome at 3 representative stages: infancy (1 month), juvenile (3 months) and adulthood (6 months) using RNA-seq techniques to establish a general overview of the differential dynamics of gene expression in the SAF and VAF, and gain further insight into functional differences in DEGs among the postnatal developmental stages and between the fat depositions.

**FIGURE 2:**
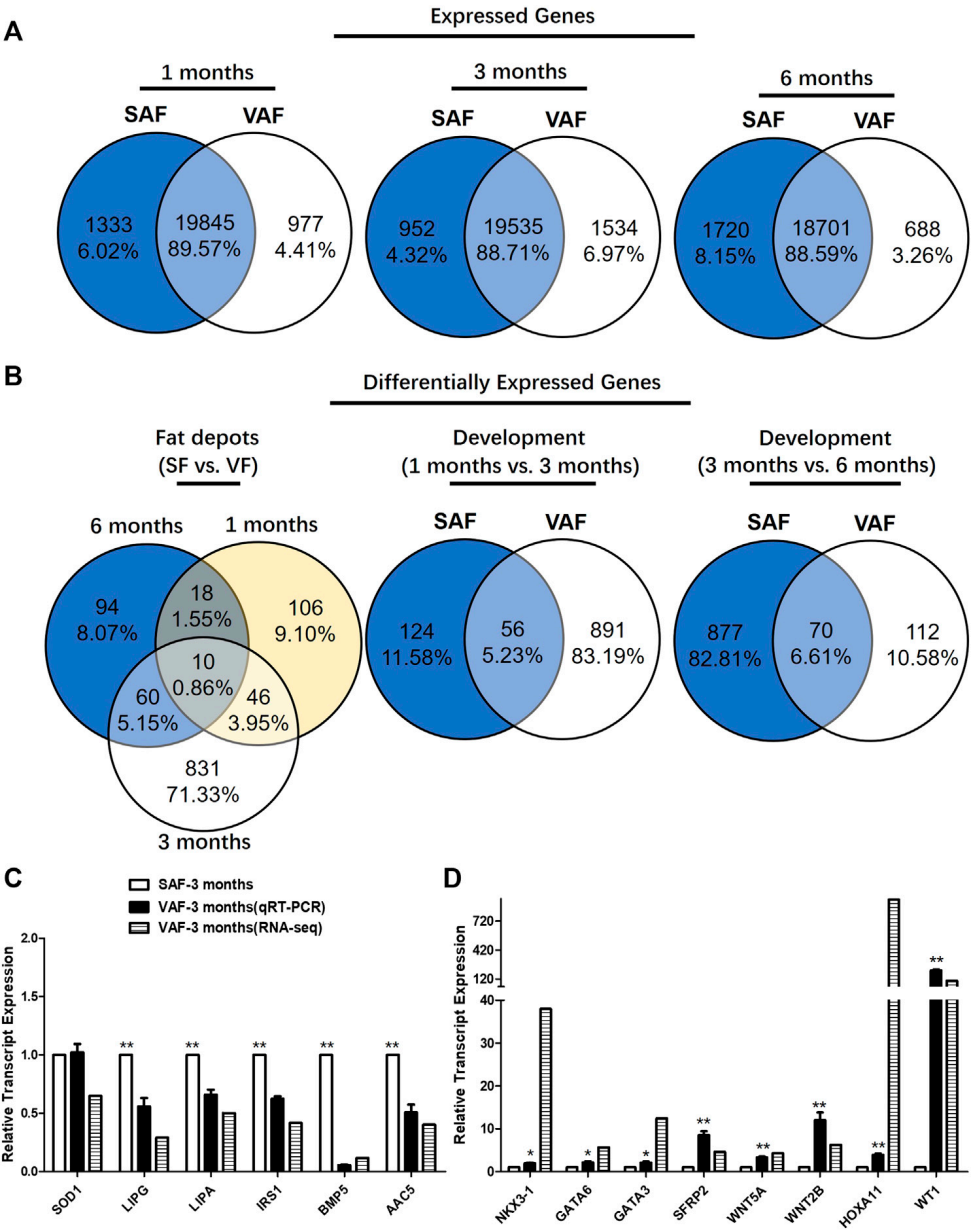
Gene identification and differentially expressed genes (DEGs) in SAF and VAF tissue from Bama pigs during the postnatal stage. **(A)** Expressed genes in each fat depot. **(B)** DEGs in each fat depot (fragments per kilobase of exon model per Million mapped fragments, FPKM) fold-change >2; *p* < 0.05). **(C)** Experimental verification of gene expression level using qRT-PCR. Relative expression levels (SAF/VAF) of 10 DEGs verified in 3 months pigs. **(D)** Experimental verification of gene expression level using qRT-PCR. Relative expression levels (SAF/VAF) of 5 DEGs verified in 3 months-old pigs (*n* = 3). ^*^Significant (*p* ≤ 0.05). ^**^Significant (*p* ≤ 0.01).

SAF and VAF samples from pig were isolated from 1-, 3- and 6 months-old pigs to profile spatiotemporal changes of the adipose transcriptome by RNA-seq using 3 replicates. A statistical table for raw and clean data sets of all RNA-seq libraries were listed in Supplementary Table S4. About 45–60 million sequencing raw reads were obtained for the 9 SAF and 9 VAF libraries respectively. ∼90% of the total clean reads were uniquely mapped to swine genome sequences in the samples (Supplementary Table S5). Using the Htseq method (Wen, 2017), the number of genes was calculated and expression data were standardized by FPKM. There were ∼23,600 expressed genes (normalized read count >3 in at least one sample) were identified, about 88.6–89.6% were detected in both fat depots (Figure 2A).

### Analysis of Consistency Within Samples

Although there is little difference among individuals in the SAF and VAF with each group, samples from each may differ in hereditary characters. Comparing the consistency within samples, those that deviated too much from the norm could be excluded from subsequent screening of DEGs, making analysis more reliable and credible. Sample correlation analysis showed that, except for VAF6-3, they were more consistent in each group. VAF6-3 deviated too much from the other VAF6 samples by using Pearson correlation coefficient (*p* < 0.65), so it was excluded from screening (results are shown in Supplementary Figure S1).

### Spatiotemporal Expression of Subcutaneous Fat and Visceral Fat Adipose-Associated Genes

There were significantly DEGs with clear fat depots, and/or age-based expression patterns. Of them, 1165 DEGs were differentially expressed between the SAF and VAF (Figure 2B). There were 180 DEGs between SAF and VAF depots at 1 month, 947 at 3 months and 182 at 6 months. Only 10 (0.86%) DEGs were shared between the SAF and VAF. Within each fat depot, there were 180 and 947 (1 vs. 3 months), 947 and 182 (3 vs. 6 months) age-based DEGs in the SAF and VAF depots, respectively, with only 56 (5.23%) and 70 (6.61%) being shared. The upregulated and downregulated genes are given in Supplementary Tables S6–S12. In each comparison, 50% were upregulated in 1 month compared to 3 months SAF; and 71% were upregulated in 3 compared to 6 months SAFs; 28% were upregulated in 1 month VAF compared to 3 months VAF; 69% were upregulated in 3 compared to 6 months VAF; 47% were upregulated in 1 month SAF compared to 1 month VAF; 23% were upregulated in 3 months SAF compared to 3 months VAF; 45% were upregulated in 6 months SAF compared to 6 months VAF.

For gene expression levels by RNA-seq, a quantitative real-time polymerase chain reaction (Qrt-PCR) was used in determining expression levels of the genes associated with lipid metabolism and the hippo signaling pathway. Regarding the Qrt-PCR used to validate relative gene expression of the 14 selected mRNAs, it is shown in Figures 2C,D that *lipase, endothelial* (*LIPG*), *lipase A, lysosomal acid, cholesterol esterase* (*LIPA*), *insulin receptor substrate 1*(*IRS1*), *bone morphogenetic protein 5* (*BMP5*), *acetoacetyl-CoA synthetase* (*AACS*) were upregulated and the expression of *NK3 homeobox 1* (*NKX3-1*), *transcription factor GATA-6* (*GATA6*), *GATA binding protein 3* (*GATA3*), *secreted frizzled-related protein 2* (*SFRP2*), *wingless-type MMTV integration site family, member 5A* (*WNT5A*), *wingless-type MMTV integration site family, member 2B* (*WNT2B*), *homeobox A11* (*HOXA11*) *and Wilms tumor 1* (*WT1*) decreased in SAF at 3 months. The tendency for changes was consistent between the RNA-seq and qRT-PCR results, except for *SOD1*. This indicates that their gene regulation networks are developmentally distinct regarding fat deposition, suggesting different functioning with age.

### Functional Enrichment Analysis

**FIGURE 3:**
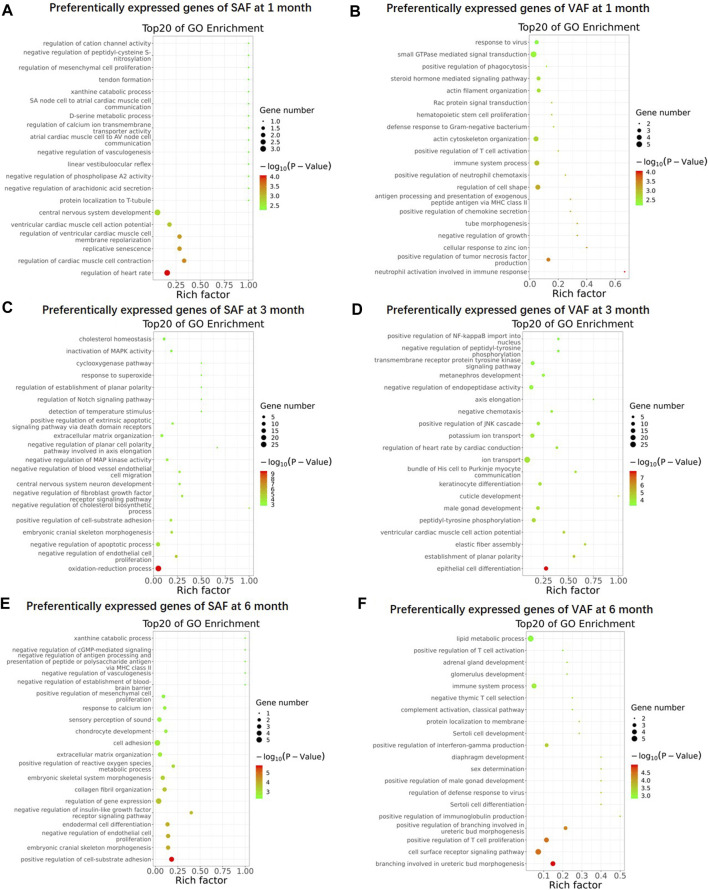
Gene Ontology (GO) enrichment of upregulated genes. GO enrichment of upregulated genes in 1 month SAF **(A)**, 1 month-old VAF **(B)**, 3 months SAF **(C)**, 3 months-old VAF **(D)**, 6 months SAF **(E)**, 6 months VAF **(F)** groups. The X-axis provides the richness factor, calculated by dividing the upregulated gene number in a given GO term by the total gene number in the term of genome. The size and color of the bubbles represent gene number and enrichment significance according to hypergeometric testing, respectively.

To gain an overview on the functions of the DEGs, we carried out Gene Ontology (GO) enrichment analysis between SAF and VAF at the same developmental stage, and identified 243, 359, 332 and 455 significantly enriched GO terms for upregulated DEGs in 1-, 3- and 6 months SAF, as also in 1-, 3- and 6 months VAF, respectively (Supplementary Tables S13–S18). Figure 3 shows the top 20 most significantly enriched GO items in each group. At 1 month, GO terms including “central nervous system development” and “regulation of heart rate” were significantly enriched in upregulated DEGs in SAF (Figure 3A); GO terms including “regulation of cell shape,” “immune system process” and “actin cytoskeleton organization” were significantly enriched in upregulated DEGs in VAF (Figure 3B). At 3 months, GO terms including “oxidation-reduction process,” “negative regulation of apoptotic process,” “cholesterol homeostasis” and “lipid metabolic process” were significantly enriched in upregulated DEGs in SAF (Figure 3C); GO terms included “epithelial cell differentiation,” “male gonad development,” “ion transport” and “positive regulation of JNK cascade” were significantly enriched in the upregulated DEGs in VAF (Figure 3D); at 6 months, GO terms including “positive regulation of cell-substrate adhesion,” “regulation of gene expression,” “cell adhesion” and “collagen fibril organization” were significantly enriched in upregulated DEGs in SAF (Figure 3E); GO terms including “cell surface receptor signaling pathway” and “immune system process” were significantly enriched in upregulated DEGs in VAF (Figure 3F).

KEGG analysis of the DEGs obtained significantly enriched pathways for upregulated genes in 1-, 3- and 6 months SAF or VAF groups (Supplementary Tables S19–S21). The enriched pathways between SAF and VAF at 1 and 6 months mainly related to diseases (Supplementary Tables S19, S21). At 3 months, pathways included “oxidative phosphorylation,” “TNF signaling pathway,” “PI3K-Akt signaling pathway,” “ECM-receptor interaction” and “focal adhesion” were significantly enriched in upregulated DEGs in SAF (Supplementary Table S20); pathways including “Hippo signaling pathway,” “thyroid hormone synthesis,” “oxytocin signaling pathway,” “calcium signaling pathway,” “focal adhesion,” “regulation of actin cytoskeleton” and “MAPK signaling pathway” were significantly enriched in upregulated DEGs in VAF (Supplementary Table S20). Deposit-specific DEGs enrichment in the lipid metabolism (SAF-specific), sexual development (VAF-specific), hormone signaling pathway (VAF-specific) and the hippo signaling pathway (VAF-specific) at 3 months are shown in Table 1.

**TABLE 1:** Deposit-specific DEGs enrichment in the lipid metabolic process (SAF-specific), sexual development (VAF-specific), hormone signaling pathway (VAF-specific) and Hippo signaling pathway (VAF-specific) at 3 months.

Gene ID	Gene symbol	Gene title		log2FC (SF/VAF)	*p*-value	FDR
SAF-specific DEGs enrichment in the lipid metabolic process						
448,985	CYP7A1	cytochrome P450, family 7, subfamily A, polypeptide 1		3.91	0	0
100,157,318	APOD	apolipoprotein D-like		3.03	0	0
396,832	LEP	leptin		2.18	0	0
100,516,456	LIPG	lipase, endothelial		1.78	0	0
100,155,736	AACS	acetoacetyl-CoA synthetase		1.32	0	0.01
100,142,668	APOE	apolipoprotein E		1.53	0	0
404,693	IRS1	insulin receptor substrate 1		1.26	0	0.05
397,576	CAV1	caveolin 1, caveolae protein, 22 kDa		1.03	0	0.03
100,156,545	LIPA	lipase A, lysosomal acid, cholesterol esterase		1.00	0	0.04
100,512,686	TWIST1	twist basic helix-loop-helix transcription factor 1		0.91	0	0.02
VAF-specific DEGs enrichment in sexual development						
100,521,236	HOXA11		homeobox A11	−9.88	0	0
397,338	WT1		Wilms tumor 1	−6.66	0	0
100,738,625	NKX3-1		NK3 homeobox 1	−5.25	0	0.02
733,631	GATA3		GATA binding protein 3	−3.64	0	0
100,520,560	WNT2B		wingless-type MMTV integration site family, member 2B	−2.65	0	0
397,600	GATA-6		transcription factor GATA-6	−2.50	0	0
100,516,027	SFRP2		secreted frizzled-related protein 2	−2.21	0	0.02
100,627,056	WNT5A		wingless-type MMTV integration site family, member 5A	−2.09	0	0
100,155,189	TESC		tescalcin	−1.12	0	0.01
VAF-specific DEGs enrichment in the hormone signaling pathway						
1.01E+08	KCNN4		potassium intermediate/small conductance calcium-activated channel, subfamily N, member 4	−8.84	0	0.01
1E+08	ASGR2		asialoglycoprotein receptor 2	−7.29	0	0.04
1E+08	CACNG6		calcium channel, voltage-dependent, gamma subunit 6	−6.66	0	0.01
396,828	KCNMB1		potassium large conductance calcium-activated channel, subfamily M, beta member 1	−4.56	0	0.01
397,081	PLA2G4F		phospholipase A2, group IVF	−4.43	0	0.04
1.01E+08	ADCY8		adenylate cyclase 8 (brain)	−4.26	0	0
1.01E+08	CACNA1D		calcium channel, voltage-dependent, L type, alpha 1D subunit	−3.73	0	0
396,898	FXYD2		FXYD domain containing ion transport regulator 2	−3.52	0	0
396,856	MYL9		myosin, light chain 9, regulatory	−3.31	0	0
1E+08	CACNA2D2		calcium channel, voltage-dependent, alpha 2/delta subunit 2	−3.25	0	0.01
1.01E+08	DUOXA2		dual oxidase maturation factor 2	−2.89	0	0
1.01E+08	MYLK		myosin light chain kinase	−2.88	0	0
1E+08	CAMK1G		calcium/calmodulin-dependent protein kinase IG	−2.83	0	0
1.01E+08	ATP1B2		ATPase, Na+/K+ transporting, beta 2 polypeptide	−1.93	0	0.01
541,593	ATP1A2		ATPase, Na+/K+ transporting, alpha 2 polypeptide	−1.89	0	0.04
1.01E+08	ATP1B1		ATPase, Na+/K+ transporting, beta 1 polypeptide	−1.76	0	0
396,848	MYL6B		myosin light chain 6B-like	−1.56	0	0.04
1.01E+08	RYR2		ryanodine receptor 2 (cardiac)	−1.51	0	0.02
VAF-specific DEGs enrichment in the Hippo signaling pathway						
1.01E+08	WWC1		WW and C2 domain containing 1	−8.84	0	0
1E+08	CDH1		cadherin 1, type 1, E-cadherin (epithelial)	−7.78	0	0
1.01E+08	WNT16		wingless-type MMTV integration site family, member 16	−7.05	0	0
1.01E+08	RASSF6		Ras association (RalGDS/AF-6) domain family member 6	−6.27	0	0
397,668	AREG		amphiregulin	−6.27	0	0
1.01E+08	DLG3		discs, large homolog 3	−3.86	0	0
1.01E+08	WNT2B		wingless-type MMTV integration site family, member 2B	−2.65	0	0
1.01E+08	PRKCZ		protein kinase C, zeta	−2.48	0	0.01
1E+08	WNT10B		wingless-type MMTV integration site family, member 10B	−2.1	0	0
1.01E+08	WNT5A		wingless-type MMTV integration site family, member 5A	−2.09	0	0
492,315	BMP7		bone morphogenetic protein 7	−2	0	0

In summary, functional enrichment analysis uncovered some unexpected results: 1) A number of the genes preferentially expressed in SAF and VAF did not overlap, especially at 3 months. 2) Enriched terms related to lipid metabolism were seen in 3 months SAF, suggesting that smaller body sizes were likely the result of their relatively high lipid metabolic rate. 3) Genes associated with sexual development were upregulated in the 3 months VAF, indicating that these pigs were at the juvenile-to-adult growth stage, consistent with the characteristics of precocious puberty in miniature pigs. 4) Genes related to the hormone signaling pathway were already highly expressed in 3-months VAF, indicating that hormone levels are different between SAF and VAF, which directly affects the metabolism of adipose tissue, as well as its accumulation and distribution. 5) Genes related to the Hippo signaling pathway were upregulated in 3 months VAF, suggesting it is important in regulating the balance of cell proliferation and apoptosis regarding organ and tissue size at 3 months.

### Short Time-Series Expression Miner Analysis

**FIGURE 4:**
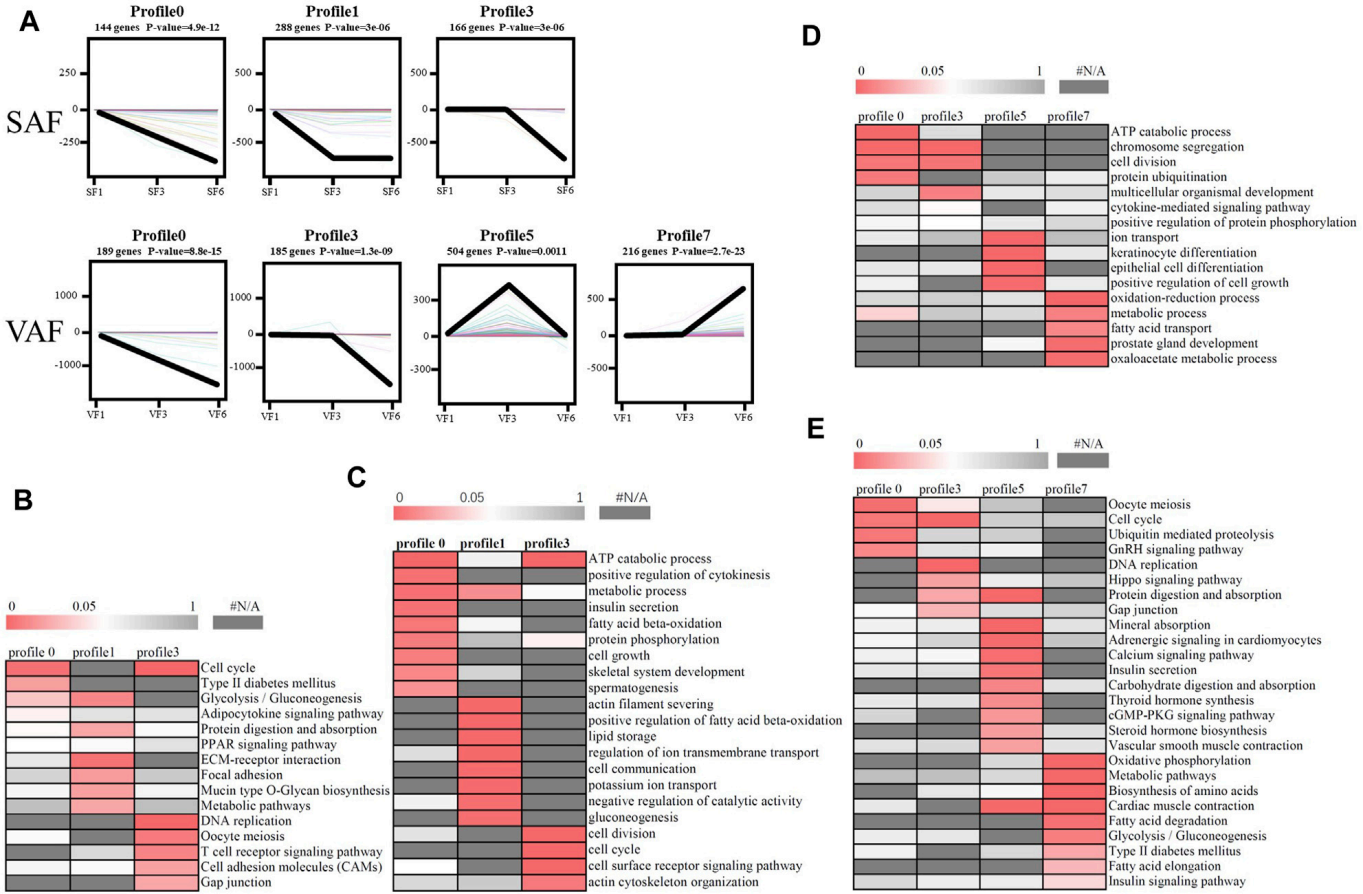
Patterns of gene expression and GO or pathway enrichment across the points in SAF and VAF. **(A)** Patterns across 3 time-points in SAF and VAF inferred by STEM analysis. In each frame, the color lines represent the expression patterns of each gene, whereas the black line represents the expression tendency of all the genes. The number of genes belonging to each pattern was labeled above the frame. **(B)** GO enrichment analysis of 3 significant clusters in SAF. **(C)** KEGG enrichment analysis of 3 significant clusters in SAF. **(D)** GO enrichment analysis of 4 significant clusters in VAF. **(E)** KEGG enrichment analysis of 4 significant clusters in VAF. The significance of the most represented GO-slims in each main cluster is indicated by *p*-value. The red areas represented the significant *p*-value, wheaeas the dark gray represented the nonsignificant values.

According to STEM analysis, all DGEs in SAF or VAF at the 3 ages were classified into 8 clusters according to their degree of expression. Among them, the *p* values of 3 clusters for SAF and 4 clusters for VAF were statistically significant (Figure 4A). The number of DGEs in the 3 clusters for SAF ranged from 144 in cluster 0 to 288 in cluster 1, while these in the 4 clusters for VAF ranged from 185 in cluster 3 to 504 in cluster 5.

**FIGURE 5:**
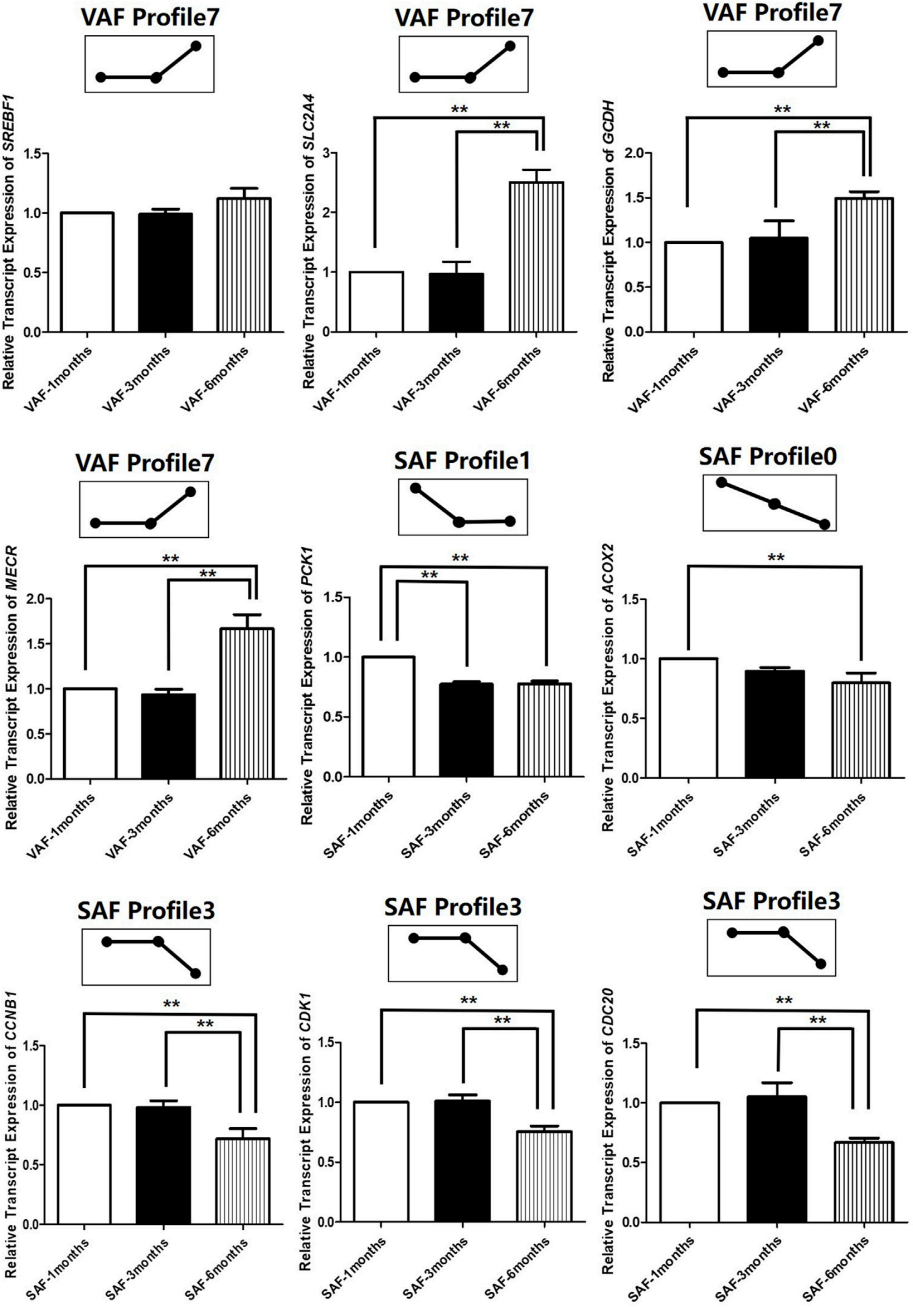
Experimental verification of gene expression level in across 3 time-points in SAF and VAF using qRT-PCR(*n* = 3). A total of 9 genes in profile 7 of VAF and profile 0, 1 and 3 of SAF were randomly selected for qRT-PCR verification. ^**^Difference is extremely significant (*p* ≤ 0.01).

To determine the significance of the transcriptional changes in each group, GO and KEGG classifications were implemented for the genes belonging to the overrepresented profiles. In SAF, genes involved in lipid storage, the PPAR signaling pathway was overrepresented in Profile 1, whereas genes involved in cell division and cycle were enriched in Profile 0 and 3 (Figures 4B,C; Supplementary Table S22). In VAF, however, the genes involved in the hippo signaling pathway were enriched in Profile 3 (Figures 4D,E; Supplementary Table S23), where expression stays the same at 1 and 3 months, but decreased at 6 months (Figure 4A), suggesting that these genes are key in regulating the size of organs and tissues during the early phase. The genes involved in the GnRH signaling pathway were enriched in Profile 0 (Figures 4D,E; Supplementary Table S23), where expression gradually decreased with age, indicating genes involved in sexual development starts at early stage (Figure 4A). In profile 5, the overrepresented GO and pathway included insulin secretion, thyroid and steroid hormone synthesis (Figures 4D,E; Supplementary Table S23). Expression levels of these genes peaked at 3 months and subsequently decreased (Figure 4A). Genes involved in lipid metabolism were enriched in profile 7(Figures 4D,E; Supplementary Table S23), where expression was lower and then peaked at 6 months (Figure 4A). These results suggested a variation in genes and pathways between SAF and VAF. Obviously, the differentiation in gene expression patterns between the two deposits corresponded to their distinct responses in sexual development, hormone signaling pathways, lipid metabolism, as well as the hippo signaling pathway. A total of 9 genes in profile 7 of VAF and profile 0, 1 and 3 of SAF were randomly selected for Qrt-PCR verification (Figure 5); the RNA-seq and Qrt-PCR patterns were consistent.

### Molecular Network in Pig Adipose Tissues Potentially Regulates Tissue Deposit- Differences

**FIGURE 6:**
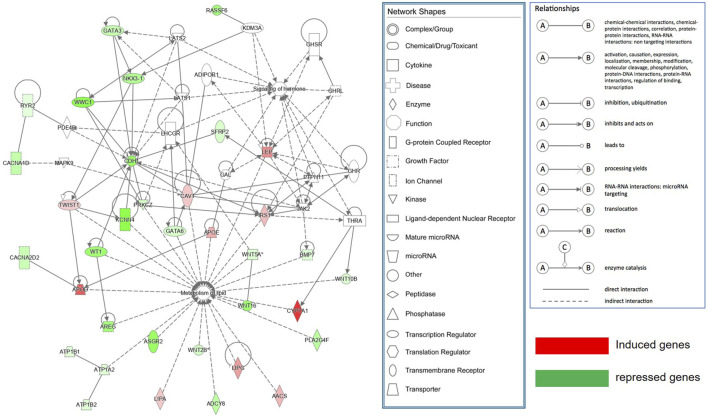
Molecular networks functionally associated with lipid metabolism and hormone signaling identified by Ingenuity Pathway Analysis (IPA).

The DEGs (log2FC >0.585 or <-0.585; ii), FDR <0.05 between SAF-3 and VAF-3 groups were used to construct a gene interaction network in the IPA system, Figure 6 showing that 35 DEGs were found. Using IPA software, function analysis of these genes showed that the most important functions of this network consisted of lipid metabolism and hormone signaling.

## Discussion

Many factors affect fat deposition, including genetics, nutrition, exercise, location of deposition, age, etc., (Lee et al., 2013; Bosello and Vanzo, 2021). Location and age are important factors affecting fat accumulation and obesity-related diseases. Miniature pigs are similar to humans in physiological characteristics, making them suitable for medical research. Our object was to study the histological differences of SAT and VAT cells at 3 ages (1, 3 and 6 months), to the follow them through development. At 1 month they have been weaned, the diet structure changes, and the organism also changes, making them the fastest growth period. At 3 months, they reach sexual maturity, the endocrine levels of hormones and other hormones change greatly, and organs are still developing. At 6 months of age, the pigs are basically mature and fully developed.

The biological functions of the adipocytes are not only being inert storage depots releasing fuel as fatty acids and glycerol in time of fasting or starvation, but also endocrine glands secreting important hormones, cytokines, vasoactive substances, and other peptides (Kahn and Flier, 2000). It is reported that the association of insulin resistance (IR) with obesity involves more than fat mass per se, including adipose location and the degree of dysfunction associated with it (Westphal, 2008). Regional differences exist in the morphological characteristics and metabolic functions of adipose tissue (Lee et al., 2013). Our findings of the adipocyte volume of SAT and VAT increasing significantly with age, being significantly larger in VAT than SAT at 3 months, consistent with available data (Hoffstedt et al., 2010). In addition, the metabolic effects of adipose depots depend on their location and whether they are characterized as VAF or SAF (Westphal, 2008). Centrally located VAT has more influence on IR than does centrally located SAT (Westphal, 2008). Fat cell size is also strongly correlated with molecular level of adipose tissue homeostasis and IR. Small fat cells are the “buffer pool” of human fat, with higher insulin sensitivity and higher absorption rate of TG and FFA, which are considered to have a protective effect (Pausova et al., 2010). However, with the gradual increase of adipocyte volume, these small adipocytes gradually changed into larger ones, with the function of IR restrained to the anti-lipopolysis effect of insulin and possessing high rate lipopolysis (Pausova et al., 2010). Several researches reported that VAT containing more fat cells with large size has stronger decomposition metabolic activity than SAT fat cells (Hoffstedt et al., 2010; Pausova et al., 2010; Lima-Martínez et al., 2013). Therefore, the total amount of VAT is generally considered to be closely associated to IR (Hoffstedt et al., 2010).

The metabolic differences of adipose tissues in different parts are mainly: lipolysis, triglyceride synthesis and storage, and also differences in adipokines secreted (Lee et al., 2013). To understand the molecular mechanism of SAF and VAF deposition differences with age, we used RNA-Seq to study the transcriptional expression of SAF and VAF at the 3 ages, and mechanisms behind then. Consistent with phenotypic differences, the most DEGs were found in VAF and SF at 3 months. Further functional enrichment analysis of DEGs in adipose tissue of VAF and SAF showed that the expression levels of genes related to lipid metabolism and cholesterol dynamic balance, which were significantly higher in SAF than IN VAF, including *APOE*(*Apolipoprotein E*), *LEPG* (*lipase, olease*), *AACS*, *APOD* (*Apolipoprotein D-like*), *CYP741* (*Cytochrome P450, Family 7, Subfamily A, Polypeptide 1*), *Caveolin 1*(*Caveolin 1, Caveolae protein, 22kDa*), and *CYP7A1* (*Cytochrome P450, Family 7, Subfamily A, Polypeptide 1*), DGEs involved in positive regulation of fatty acid β oxidation; *IRS1* and *TWIST1* (*Twist Basic helix-loop-Helix transcription factor 1*) were also highly expressed in SAF at 3 months. APOE is an enzyme involved in the activation of hydrolyzed fat and the transformation and metabolism of lipoprotein. Its concentration is positively correlated with plasma triglyceride content, and is an apolipoprotein that regulates lipid metabolism and cholesterol balance. It has an anti-atherosclerosis effect by inhibiting the formation of foam cells (Getz and Reardon, 2018). APOD is an apolipoprotein in plasma HDL and VHDL, and has the function of binding and transporting lipids by associating with APOA-I, and lecithin Cholesterol acyl transferase (LCAT) forms a “cholesterol ester transport complex” that helps regulate cholesterol metabolism (Thomas et al., 2003). *Endothelial lipase* (*EL*) gene, a member of lipase family, is expressed at a low level in normal endothelial cells, and its expression being significantly increased by various atherosclerosis (AS) factors (McCoy et al., 2002), which suggests that at 3 months, the cholesterol dynamic balance of SAT is higher than that of VAT, which is associated with higher TG and FFA absorbability and higher small-volume fat cells in SAT (Pausova et al., 2010). Other studies have shown that differences in expression of *HSL* and *perilipin*, encoding a lipid droplet protein that regulates lipolysis, in omental adipose tissue and SAT may be one of the molecular mechanisms underlying the differences between sites of lipolysis (Arner, 1995; Wang et al., 2003). However, the expression levels of these two genes were not significantly different between SAT and VAT, which may be due to the different species and parts of the subjects examined.

Adipose tissue is not only an energy storage organ, but has the potential to release a large number of peptide hormones and active adipocytes through autocrine, paracrine and endocrine forms. Adipokines secreted by SAF and VAF are involved in a number of physiological and pathological processes, including appetite and energy metabolism, IR, reproductive and endocrine system, bone metabolism, immunity and inflammation (Al-Gareeb et al., 2016); the characteristics of these adipokines are different (De Oliveira Leal and Mafra, 2013). Therefore, the study the molecular mechanism of functional differences in adipose tissue of different parts in the prevention and treatment of obesity and obesity-related diseases is important. We found a significant difference in insulin sensitivity between SAT and VAT at 3 months. Insufficient secretion and action of insulin are two important factors leading to IR and diabetes. IR has decreased efficiency of insulin in promoting glucose uptake and utilization for several reasons, and the body produces hyperinsulinemia through compensatory secretion of excessive insulin to maintain the stability of blood glucose (Lebovitz, 2001). We identified *Leptin* (*LEP*) and *IRS1* genes associated with insulin response through analysis of DEGs in SAT and VAT at 3 months. *IRS1* expression in SAT was significantly higher than in VAT. However, the expression levels of 9 genes related to insulin secretion in VAT were significantly higher than those in SAT, such as *ATPase, Na* ^*+*^ */K* ^*+*^ *buffer, beta 2 polypeptide, ATP1B2*; *ATPase, Na* ^*+*^ */K* ^*+*^ *buffer, beta 1 polypeptide, ATP1B1*; *ATPase, Na* ^*+*^ */K* ^*+*^ *buffer, Alpha 2 polypeptide, ATP1A2; Adenylate cyclase 8* (*brain*)*, ADCY8*; *Ryanodine receptor 2* (*Cardiac*)*, RYR2*; *FXYD domain containing ion Transport Regulator 2, FXYD2*, etc., IRS1 is an important molecule in intracellular insulin signaling (Tamemoto et al., 1994). Leptin is a protein-like circulating factor, mainly involved in body weight regulation by reducing food intake (Halaas et al., 1995), increasing energy consumption (Pelleymounter et al., 1995), strengthening substance metabolism and other ways. The mechanism is through receptors on the target cell membrane and the corresponding signal transduction system. Signal transduction through bidirectional activated kinase J tyrosine protein kinase (JAK) and its transduction and the transcriptional activation protein (STAT) pathway affects the secretion of neuroendocrine hormones, such as neuropeptide Y (NPY), resulting in decreased appetite, increased energy consumption and loss of weight (Bjorbæk and Kahn, 2004). Powe et al. (2019) reported that LEP was significantly positively correlated with insulin response, insulin sensitivity and fat mass during the first trimester. LEP has a bidirectional effect on β cell function and glucose-stimulated insulin secretion (Kulkarni et al., 1997; Highman et al., 1998; Covey et al., 2006). We found that the expression of ATPase gene related to insulin secretion pathway was significantly higher in VAT than in SAT. ATP is a major signal of pancreatic IR (Ye, 2021). In obesity, ATP overproduction promotes systemic IR through a several mechanisms, such as inhibition of AMPK, Mtor induction, hyperinsulinemia, hyper glucagon and mitochondrial dysfunction. Preventing overproduction is a key strategy for insulin sensitization, which suggests that SAT is significantly more responsive and sensitive to insulin than VAT at 3 months, and the molecular basis of VAT’s higher metabolic activity determines that this site is closely related to IR (Hardy et al., 2012).

We found that some genes involved in gonadal development and endocrine function were significantly different in SAF and VAF expression at 3 months. Through DEGs pathway analysis, expression of 9 genes related to male sexual development in 3 months VAF was significantly higher than in 3 months SAF, such as *Nkx3-1*, *GATA-6*, *transcription factor GATA-3* (*GATA-3*), *SFRP2*, etc. *Nkx3.1* gene is one of the new NK family homeobox genes that has a regulatory role in embryonic development (Tanaka et al., 2000), and is one of the key regulatory genes involved in prostatic genesis. Expression of *Nkx3.1* is highly specific to prostatic epithelium (Gelmann et al., 2003), and in the development of embryos, its expression is parallel to the level of androgens. Studies on adult male mice showed that its mRNA expression is significantly increased during sexual maturation, but significantly decreased after castration, *Nkx3.1* being maintained at high expression in a time and concentration dependent manner by androgen (Prescott et al., 1998). GATA-3 and GATA-6 are members of a group of zinc finger-containing transcriptional regulatory factors important in the mammalian reproductive system, including sex determination and reproductive function regulation (De Rooij, 1998; Tremblay and Viger, 2001). GATA-3 is mainly expressed in the pituitary gland, whereas Gata-6 is mainly expressed in endocrine organs and heart, and is highly expressed in testis of human and other vertebrates. It affects the growth and development of testis of mice, promotes the proliferation of stromal and spermatogonial cells, and synthesizes androgens (Bennett et al., 2013; Convissar et al., 2015; Jahromi et al., 2016). The data shows that pigs are in the stage of puberty and sexual maturity. SAF and VAF are important in regulating gonad development in male pigs and there are obvious molecular differences. The results of expression trend analysis also supported this conclusion. We found that genes related to the GnRH signaling pathway were enriched in VAF significant expression trend Profile 0. With increasing age, expression of these genes was highest at 1 month, and gradually decreased by 3 and 6 months. Gonad development and sexual maturation initiation in mammals are regulated by the hypothalamic-pituitary-gonad axis. GnRH, a hormone secreted by the hypothalamus to regulate reproductive function, is critical in sexual maturation initiation. These results indicate that genes involved in pituitary-gonad axis in pigs are highly expressed in the early stage of VAF, which to some extent reveals the molecular mechanism of precocity and reproduction.

We also found that thyroid hormone synthesis and the prolactin signaling pathway related genes were highly expressed in 3 months VAF. Gene expression trend analysis also showed that, in Profile5, gene expression of insulin secretion, oxytocin signaling, thyroid hormone synthesis and steroid hormone biology pathways peaked at 3 months, being lower at 1 and 6 months. Oxytocin helps to reduce weight by reducing food intake and increasing energy expenditure and fat breakdown. Oxytocin signaling is closely related to visceral fat deposition, such that animals without it show significantly increased abdominal fat, the weight of fat pads in perirenal, mesenteric and epididymal regions being significantly increased (Wu et al., 2012). Obesity is closely related to thyroid function, and produces an important regulatory neurohormone that affects cell differentiation and development on one hand, but on the other hand it regulates various metabolic functions in the body, especially of fat and blood sugar. Decomposition of thyroid hormone on fat and blood sugar is often stronger than the synthesis effect of fat and blood sugar, which may be an important reason for the high metabolic activity of VAT (Pausova et al., 2010). The data suggest that prolactin signaling and thyroxine synthesis signaling pathways help regulate VAF energy balance; in particular, during sexual maturation, VAF adipose tissue development is closely related to reproductive and endocrine functions, including insulin secretion, gonadal axis development and the thyroid hormone, etc.

Because miniature pigs are small body size, one can determine how accurately control of the organ size is in maintaining tissue homeostasis and normal body function, issues that are being widely studies. We found that 11 genes involved in the Hippo signaling pathway were significantly higher in 3-months VAF than in SAF, including *bone Morphogenetic protein 7*(*BMP7*), *wingless-type MMTV integration site family member 16* (*WNT16*), Ras association (*RalGDS/AF-6*) *domain family member 6*(*RASSF6*), *amphiregulin* (*AREG*), *WNT5A, wingless-type MMTV integration site family, member 10B*(*WNT10B*), *WNT2B*, *WW and C2 domain containing 1* (*WWC1*), *discs, large homolog 3*(*DLG3*), *Protein kinase Cζ Zeta* (*PRKCZ*), etc., BMP7 is an important member of the Transforming growth factor-β (TGF-β) family (Bragdon et al., 2011), and is a secreted multifunctional protein. The heterodimer of BMP2/BMP7 and BMP4/BMP7 involved in the formation of BMP2/BMP7 and BMP4/BMP7 have good osteogenic bioactivity; it is prominent in promoting osteogenesis and cartilage. The Wingless-type MMTV Integration site family member (WNT) is a class of proteins that are important in growth and development, as well as in physiological and pathological processes. It can initiate intracellular WNT signal transduction pathways and conduct growth stimulus signals; it is also involved in different developmental mechanisms including cell proliferation, polarity, movement, differentiation, survival, self-renewal and calcium balance (Logan and Nusse, 2004; Mohammed et al., 2016; Doumpas et al., 2019). WNT5A, WNT16, WNT10B and WNT2B are important members of the WNT family, with their signal transduction pathways falling into classical pathways that determine cell fate and non-classical pathways that control cell movement and tissue polarity (Mohammed et al., 2016). The classical signaling pathway is involved in skeletal muscle development, affecting the fusion of myoblast cells (Suzuki et al., 2018) and mediating the regulation of Yes-associated protein (YAP) on bone homeostasis (Pan et al., 2018). In conclusion, VAT can be important role in bone and muscle development of Bama pigs by expressing BMP and WNT protein genes and activating the WNT signaling pathway. The Hippo signaling pathway is a growth mechanism that precisely controls organ size and maintains tissue homeostasis by balancing cell proliferation and apoptosis. Its core member is composed of the upstream kinase complex mammalian Sterile 20-like kinase (MST) MPS One Binder kinase, MOB) and the downstream transcription complex-YAP/ PDZ-binding motif, YAP/ TAZ-TEADS regulate cell fate by controlling the expression of time- and space-specific target genes (Zhou et al., 2016). He et al. (2020) first discovered *YAP* and *nuclear receptor subfamily 4, Group A, member 1* (*NR4A1*) in the Hippo signaling pathway, which can precisely determine the balance between cell proliferation and apoptosis to control organ size and maintain tissue homeostasis (He et al., 2020). Based on the high expression of genes involved in the Hippo signaling pathway in 3 months VAT, we speculate that adipose tissue may be helpful in regulating the balance of cell proliferation and apoptosis controlling organ and tissue size through the Hippo and the WNT signaling pathways, and VAF could be more important than SAF. The functions and molecular mechanisms of the Hippo pathway and WNT adipose tissue expression related genes in pigs are worth further study.

IPA molecular network analysis has also shown that there are 35 DEGs involved in metabolism of lipid and signaling of hormones (Figure 6). 11 genes involved in the Hippo signaling pathway analyzed by KEGG were also included in this network, indicating that adipose metabolism and its endocrine function have a role in controlling the organ size. It is also suggested that regional differences of adipose not only influence body metabolism, but affect growth and development, regulating organ size. Those findings are consistent with the findings during *Drosophila* development where fat restricts organ size via the Salvador/Warts/Hippo pathway (Bennett and Harvey, 2006). STEM analysis integrated with the Gene Ontology also was used to explore the expression pattern of the DEGs in SAF and VAF among the three groups of pigs. Expression levels of the genes related to the cell cycle and the Hippo signaling pathway were downregulated in VAF as the pigs reached 6 months, including 15 genes (Supplementary Table S23). The findings suggested that VAF is involved in the regulation of organ size at the early stages of postnatal development. This novel finding demonstrates that adipose tissues genes expressed in different regions are closely associated with regulation of the size of a miniature pig. Further molecular validation is needed to elucidate the exact function of relevant adipose tissue genes in controlling the body size.

## Conclusion

Through transcriptional analysis of the genes expressed in the SAF and VAF tissues of 1-, 3- and 6 months pigs, we identified the genes and pathways that are preferentially expressed in SAF or VAF tissues. These DGEs were functionally related to sexual development, hormone signaling pathways, lipid metabolism and the hippo signaling pathway, suggesting the importance of the signaling pathways contributing to significant regional and developmental stage differences. The findings highlight the essential functions of the molecular network in adipose tissues that potentially regulate adipose tissue deposition differences. Combining the molecular network and DGEs expression analysis, a molecular network that includes 35 DEGs involved in lipid metabolism and hormone signaling has been constructed in the IPA system. Our findings will help in further studies with genes relevant these differences between SAF and VAF during postnatal development. The data provides greater insight into the molecular mechanism of heterogeneity between SAF and VAF critical for health and disease processes of perhaps other living beings by the regulation of energy metabolism and endocrine function.

## Data Availability

The datasets presented in this study can be found in online repositories. The names of the repository/repositories and accession number(s) can be found in the article/Supplementary Material.

## Abbreviations

DEGs, differentially expressed genes; FPKM, fragments per kilobase of exon model per Million mapped fragments; GO, gene ontology; HSL, hormone sensitive lipase; IL-6, interleukin-6; IL-8, interleukin-8; IMF, intramuscular fat; Ips, pluripotent stem; IPA, ingenuity pathway analysis; KEGG, Kyoto Encyclopedia of Genes and Genomes; MCP-1, macrophage chemoattractant protein-1; Qrt-PCR, quantitative real-time polymerase chain reaction; RNA-Seq, RNA sequencing technologies; STEM, Short time-series expression miner; SAF, subcutaneous fat; VAF, visceral fat.
