# Supplementary material for: Comparative Transcriptome Analysis Provides Insight into Spatio-Temporal Expression Characteristics and Genetic Regulatory Network in Postnatal Developing Subcutaneous and Visceral Fat of Bama Pig
Source: Front Genet. 2022 Mar 31;13:844833. doi: 10.3389/fgene.2022.844833 (PMC9008487; doi:10.3389/fgene.2022.844833)
Supplement: Supplementary file 1 [file DataSheet1.ZIP › Supplemental Table 1-2 Primers for Real-time PCR validation.docx]

Supplemental Table 1 Primers of Real-time PCR validation for 15 DEGs between SAF and VAF in 3 months-old pigs

| GB No. | Genes | Sequence（5'-to-3') | Amplication  size（bp） |
| --- | --- | --- | --- |
| NM_001190422.1 | SOD1 | F TCGGGAGACCATTCCATCAT | 116 |
|  |  | R GCCAAACGACTTCCAGCATT |  |
| [NM_001243029.1](https://www.ncbi.nlm.nih.gov/entrez/viewer.fcgi?db=nucleotide&id=339895858) | LIPG | F CCTGTGAGATTTAACCTCCGAAC | 285 |
|  |  | R TCCCACTTTCCTCGTGTTATTG |  |
| NM_001123134.1 | LIPA | F CTCTTGGGTTTGGTGGTCTGTT | 224 |
|  |  | R TTGGGACCTTTGTCAGAGTGGT |  |
| NM_001244489.1 | IRS1 | F TGCCTGACCAGCAAGACCAT | 172 |
|  |  | R AATCATCCACCTGCATCCAAA |  |
| NM_001204901.1 | BMP5 | F CCATGATGCCAACTTTCTCAAC | 159 |
|  |  | R AATTCTGCTGCTGTCACTGCTT |  |
| [XM_021072487.1](https://www.ncbi.nlm.nih.gov/entrez/viewer.fcgi?db=nucleotide&id=1191834807) | AACS | F CACTGGGAAAGGCGAGCAA | 211 |
|  |  | R GCCGACCGTGGTGTAATAGAAG |  |
| [XM_003483391.4](https://www.ncbi.nlm.nih.gov/entrez/viewer.fcgi?db=nucleotide&id=1191834320) | NKX3-1 | F TAGGCACCTTGAGACTTATCTGCT | 209 |
|  |  | R TCTCCGTGAGCTTGAGGTTCTT |  |
| NM_001244395.1 | SFRP2 | F CCTGCAAAAACAAAAACGAGG | 255 |
|  |  | R CCAGATAGGGCGCGTTGATA |  |
| XM_013981798.2 | WNT5A | F GCAATGTCTTCCAAGTTCTTCCTA | 176 |
|  |  | R CCTTGAGAAAGTCCTGCCAGTT |  |
| XM_003125849.5 | WNT2B | F GTGAGTGGCTCCTGTACTCTGC | 286 |
|  |  | R TTTCACAACCGTCTGTCCCTT |  |
| NM_214328.2 | GATA6 | F TGCCAACTGTCACACCACGA | 262 |
|  |  | R GGAAGTATTTTTGCTGCAATCG |  |
| XM_003134850.4 | HOXA11 | F GAATACGCCATTGAGCCCG | 284 |
|  |  | R TCGGAGGAGGCGAGGTTTT |  |
| XM_021064071.1 | GATA3 | F CAACCACGTCCCGTCCTACTA | 189 |
|  |  | R GTCTTGGAGAACGGGCTGAG |  |
| NM_001243629.1 | CTSA-S | F AACCCCTATTCTTGGAACCTGAT | 123 |
|  |  | R ATTGCTCTGGGCGACTTCC |  |
| NM_001001264.1 | WT1 | F TTCATCAAACAAGAGCCGAGC | 242 |
|  |  | R GTGCTGTATCCCTGGTTGCG |  |
| NM_001206359.1 | GAPDH^a^ | F GTGAAGGTCGGAGTGAACGGA | 252 |
|  |  | R CCATTTGATGTTGGCGGGAT |  |

^a^ GAPDH is the internal control gene.

Supplemental Table 2 Primers of Real-time PCR validation for 9 genes in patterns of gene expression across three time points in SAF and VAF

| GB No. | Genes | Sequence（5'-to-3') | Amplication  size（bp） |
| --- | --- | --- | --- |
| NM_001123158.1 | PCK1 | F CAGCCTGACCAAATCCACATC | 182 |
|  |  | R TGCTCCTGAGTAATGATGACCG |  |
| XM_021069070.1 | ACOX2 | F AACAGATTGCCAAGTGGGACC | 281 |
|  |  | R GCTCCTGATGGGCACAATGAA |  |
| NM_001170768.1 | CCNB1 | F ACTGCTCTTGGAGACATCGGTA | 195 |
|  |  | R TGGTTCAGGCTCCAGTTCAG |  |
| NM_001159304.2 | CDK1 | F GGAAGGTGTTCCTAGTACTGCC | 176 |
|  |  | R TGAACTGACCAGGAGGGATAGAA |  |
| NM_001123094.1 | CDC20 | F CCAGAGGGTTACCAGAACAGAC | 149 |
|  |  | R ACCAGGTTCAGGTAGTAGTCGT |  |
| NM_214157.1 | SREBF1 | F GCTGAATAAATCCGCCGTCTTG | 134 |
|  |  | R CAGGTCCTTCAGAGACTTGCTT |  |
| NM_001128433.1 | SLC2A4 | F CTGGGAAGGAAGAAGGCAATG | 120 |
|  |  | R AATGAGGAACCGTCCAAGAATG |  |
| XM_021083716.1 | GCDH | F CGAAGAGCAGCAGCAGAAATAC | 202 |
|  |  | R CACTACAAACAGGTCAGCCACA |  |
| NM_001244082.1 | MECR | F CTTGCTCTCAACTGTGTTGGC | 193 |
|  |  | R GGTCTGGATTGTGGTCCTTCTT |  |
| NM_001206359.1 | GAPDH^a^ | F GTGAAGGTCGGAGTGAACGGA | 252 |
|  |  | R CCATTTGATGTTGGCGGGAT |  |

^a^ GAPDH is the internal control gene.
